# Peer supporters' mental health and emotional wellbeing needs: Key factors and opportunities for co‐produced training

**DOI:** 10.1111/hex.13836

**Published:** 2023-08-10

**Authors:** Laura Kane, Robert M. Portman, Judith Eberhardt, Lauren Walker, Emma‐Lily Proctor, Hannah Poulter, Catherine O'Neill

**Affiliations:** ^1^ Department of Psychology, Centre for Applied Psychological Science, School of Social Sciences, Humanities & Law Teesside University Middlesbrough UK

**Keywords:** emotional, mental health, peer support, peer supporter, recovery, training, wellbeing

## Abstract

**Introduction:**

Peer supporters are a valuable asset to mental health and support services, but their own mental health needs are often overlooked in research and practice. This study explored peer supporters' perceived challenges of maintaining their mental health and emotional wellbeing and co‐produced training needs.

**Methods:**

A qualitative approach was used to explore factors affecting peer supporters' mental health and emotional wellbeing. Semi‐structured interviews and focus groups were conducted online with 11 peer supporters across North East England.

**Results:**

A thematic analysis identified: ‘Lack of training and support’, ‘Role ambiguity’ and ‘Emotional labour’ as challenges experienced by peer supporters in relation to maintaining their mental health and emotional wellbeing. Peer supporters' own lived experiences had the potential to act as a barrier towards providing support to others. Conflict with peer ‘supportees’ sometimes negatively impacted on the peer supporter experience. Participant responses emphasised a need for person‐centred, co‐produced training.

**Conclusion:**

This work highlights the need for targeted training for peer supporters, including both role‐specific education and strategies to support their mental health and emotional wellbeing.

**Patient or Public Contribution:**

Participants were contacted and asked to provide feedback on finalised themes to ensure the analysis was congruent with their experiences, further enabling the future development of an emotional wellbeing training programme for peer supporters.

## 1 INTRODUCTION

Peer support is recognised as people with lived experience of a particular experience, life circumstance or disability, guiding others experiencing similar difficulties through mutual support.^1^, ^2^ Effective peer support provision is built upon the foundations of mutuality and shared respect.^3^ Peer support has previously been divided into three broad categories: (1) informal naturally occurring peer support which is not associated with organisations or services; (2) peer supporters working or volunteering in peer‐led programmes or services and (3) peer supporters employed in traditional health settings.^4^

Recently, there has been an increase in the implementation of peer support within mainstream mental health settings due to a transformational drive towards a recovery‐orientated approach to mental health.^2^ Countries, including the United Kingdom, now endorse peer support at a policy level by including previous service users in the development and delivery of peer support schemes.^5^ Thus, people with mental health difficulties who have previously engaged with services are subsequently provided with an opportunity to be an integral part of future service development and delivery.^6^ Peer support employed in mental health settings is positively associated with a range of recovery‐based outcomes, such as increased hopefulness,^7^ self‐esteem,^8^ self‐efficacy and self‐determination,^3^ as well as social inclusion and condition management.^9^ In addition to the individuals who are the direct recipients of peer support, peer supporters themselves report increased perceptions of a sense of purpose, self‐esteem and self‐determination.^10^ As such, the positive impact of peer support is multidirectional. Notwithstanding, studies exploring the effectiveness of peer support interventions have found little difference in clinical outcomes when comparing peer support‐assisted treatment with traditional support services.^8^, ^9^

Although peer support schemes can yield positive outcomes,^3^, ^7^, ^8^ implementing peer supporters in support settings can bring significant challenges. Peer support roles within these settings are often ill‐defined, leading to a multitude of negative implications for peer supporter wellbeing, including anxiety, frustration and a hesitancy to act.^11^, ^12^ Ill‐defined peer roles can also reduce peer supporter acceptance and integration into traditional mental health settings.^13^ Peer supporters can encounter challenges when attempting to create and maintain boundaries between themselves and those whom they support, leading to emotional distress.^13^, ^14^ The emotional labour inherent in peer support can also lead to emotional distress and exhaustion.^14^ Hence, peer supporters can experience difficulties in maintaining their wellbeing and recovery.^12^ The combination of emotional labour, role isolation and lack of emotional support can lead to a heightened burnout risk for peer supporters.^14^ Lack of supervision and support is a commonly reported problem within peer support settings.^12^, ^15^, ^16^ In addition, peer supporters often report a lack of relevant training for their role,^13^, ^17^ and where training is offered, this can be incongruent with peer support values.^18^

Collectively, the accumulated knowledge of challenges experienced by peer supporters is predominantly drawn from evaluations of peer‐based interventions which focus on the outcomes from the vantage points of the recipients of peer support and peer support organisations themselves. As such, the focus on peer supporters, and the challenges that they experience, is traditionally of a peripheral nature. More peer supporter‐centric research is required to produce a better in‐depth contextual understanding of the challenges experienced by peer supporters, and how to best overcome these. A systematic literature review^13^ has shown two common issues in peer supporter training: overprofessionalised training that is incongruent with peer support values, and a lack of role‐specific knowledge. Both can have a detrimental impact on peer supporter mental health and emotional wellbeing,^11^, ^12^, ^18^ emphasising the need for further co‐produced training opportunities^15^ that are in line with the values of peer support.^19^ Further research is needed to: (1) explore peer supporters' experiences in relation to the barriers they face as part of their role, particularly in relation to their mental health and emotional wellbeing, and (2) create a better understanding of how to address this gap in peer supporter training. Therefore, this study aimed to answer the following research question: what barriers do peer supporters experience in relation to maintaining their mental health and emotional wellbeing, and how could these be addressed through co‐produced training?

## 2 METHODS

### 2.1 Design

The study employed a qualitative design, using semi‐structured interviews and focus groups. This section is presented in line with the Consolidated Criteria for Reporting Qualitative Studies guidelines.^20^

### 2.2 Participants and procedure

Ethics approval was obtained by Teesside University's research ethics committee. Opportunistic sampling was employed.^21^ The research team contacted 33 local peer supporter organisations operating in North East England. These organisations offered support across a diverse range of contexts, including veteran support, addiction recovery and migrant support. Eleven individuals who provided peer support agreed to participate in an interview or focus group. This sample size is appropriate for small qualitative studies employing thematic analysis.^22^ All participants were provided with a participant information sheet and provided their informed consent before being interviewed. Participants were thanked post‐interview and provided with contact details of organisations offering information and support. Key participant demographic characteristics and their respective peer support settings are presented below in Table 1.

**Table 1:** Participant demographic characteristics and peer support settings.

Pseudonym	Gender	Peer support setting
Jeremy	Male	Veteran support
Louise	Female	Addiction recovery
Richard	Male	Addiction recovery
Andrew	Male	Addiction recovery
Thomas	Male	Men's mental health
Rachel	Female	Sexual violence support
Amanda	Female	Social enterprise
Paul	Male	Addiction recovery
Jason	Male	Migrant support
Lisa	Female	Migrant support
Janine	Female	Addiction recovery

An interview schedule was devised, informed by the existing literature on peer supporters, as well as the research team's expertise in this area — two members of the research team had worked previously as peer supporters. The schedule covered topics such as peer supporter roles, experiences of peer support and perceived further training and emotional support needs, and was agreed upon by the research team. Discussions within the research team as part of the development of the interview schedule covered what was already known in the literature on peer supporter training and their emotional support needs, and time was then given to the members of the research team who worked as peer supporters to add any aspects that had not been covered in the literature, but rather, had arisen as part of their own experiences. Questions included: ‘What training did you have (if any) as a peer supporter?’; ‘What has worked well in the past with regards to peer supporter training?’ and questions in relation to co‐produced training, for example, ‘Would you be interested in helping us design or deliver some training?’ Other questions focussed on challenges experienced in their role, for example, ‘What kind of challenges have you experienced as a peer supporter?’ (see Table 2 for the full interview schedule). Data collection was undertaken by four researchers experienced in conducting qualitative interviews (L. W., E. L. P., C. O. N. and H. P.). Five one‐to‐one semi‐structured interviews and one focus group were conducted and recorded via Microsoft Teams. Participants were provided with the choice of attending a focus group or semi‐structured interview, thereby ensuring they were comfortable, and that data collection was participant‐led, congruent with the values of co‐production.^19^ Using focus groups and individual interviews did not result in notable differences in the type of data collected; the same interview schedule was used across interviewees ensuring consistency in the way the data were collected, while combining focus groups with interviews enhanced the richness and quality of the data by offering participants a choice, thereby providing them with an optimised interviewing experience.^23^ Furthermore, as the data collected through both methods yielded similar findings, their trustworthiness was thereby enhanced.^23^ Interview duration ranged from 19 to 116 min (mean duration = 52 min) and the focus group lasted for 60 min. Interviews were transcribed verbatim with any personally identifiable information removed and/or replaced with pseudonyms to ensure anonymity. No financial incentives were provided for participating in this study.

**Table 2:** Interview schedule.

Order	Question
1	What brought you to become a peer supporter?
2	Was there anything particular that you wanted to get out of it?
3	Why do you believe in a peer support approach?
4	What does it offer that other approaches don't offer?
5	What kind of challenges have you experienced as a peer supporter?
6	What does peer support look like for you?
7	What kinds of things do you do?
8	Do you have a role description?
9	What does a good peer supporter look like in your organisation—what kind of characteristics do they have?
10	What training did you have (if any) as a peer supporter?
11	What has worked well in the past with regards peer support training?
12	Have you received any training specifically related to your emotional wellbeing?
13	If not, what training or support might be useful?
14	What barriers might there be to attending emotional wellbeing training as a peer supporter?
15	Any ideas on how we could overcome these barriers?
16	Would you be interested in helping us design or deliver some training?

### 2.3 Data analysis

Data were analysed thematically^24^ by members of the research team (L. K., L. W.). First, data familiarity was achieved via reading through the transcripts several times. The second step involved systematically coding interesting features within the data set and creating initial codes. Next, codes were provisionally reorganised by grouping together codes related to similar topics, leading to the creation of themes. At step 4, these themes were reviewed by each analyst on two levels: at the first level, it was examined whether the coded extracts were congruent with each theme, and at the second level, it was determined whether themes were congruent with the data. Step 5 involved further refinement and naming of the identified themes. Themes were finalised once agreement had been reached between all researchers. Lastly, themes were written up and extracts which most suitably represented the themes were included as illustrative examples. A latent approach was undertaken throughout to obtain a more extensive understanding of participants' experiences, which may not be possible at a semantic level.

## 3 RESULTS

Three key themes were identified that described challenges to peer supporters' mental and emotional wellbeing, and co‐produced training needs. These themes are inherently interwoven and likely cumulative; each theme representing a different manifestation of emotionally taxing experiences for peer supporters. The first theme, ‘Insufficient support for peer supporters’, highlighted the lack of appropriate training provided to peer supporters and emphasised the need for additional focus on their own wellbeing due to the emotional labour of the role. This theme also highlighted how peer supporters perceived that their supervision and social support needs were not consistently met by their organisations. The second theme, ‘Where does peer support begin and end?’, was identified as an unintended negative consequence of the flexible, person‐centred and reciprocal nature of peer support provision. Role ambiguity was associated with difficulties in implementing and maintaining boundaries. The third theme, ‘Emotional investment leading to exhaustion’, highlighted the ways in which peer supporter roles can contribute towards emotional exhaustion and distress. The theme also details how exposure to traumatic disclosures from peers can be detrimental to peer supporters' mental health and emotional wellbeing.

### 3.1 Theme 1: Insufficient support for peer supporters

This theme highlighted peer supporters' perceived lack of receiving appropriate training and the challenges this presented in relation to their mental health and emotional wellbeing. Some peer supporters discussed barriers to accessing training with organisations and individual peer supporters held different views on the suitability of the content of the training offered. Peer supporters discussed experiencing minimal social support and supervision in some services, which led to emotional distress, exhaustion and frustration.

Jeremy discussed his training experience: ‘Coming into the role it was just really, you know, covering the basics; DBS check; safeguarding; Prevent. But as a way of you know, training for the role, none’. Although Jeremy discussed that he had received some training, he highlighted the lack of role‐specific training offered by the organisation.

Participants also emphasised the importance of tailoring training opportunities to the population and the individual barriers that certain subsections of the peer support community may experience. Rachel, a peer supporter for victims of sexual violence, described such an experience:

We were supposed to maybe have some from [organisation] of part of their peer support training, or training for some of our women if they wanted to go forward to do the peer support leader program, but some of our women didn't want to do that because it was facilitated by a man and there was no other option to do that. So, I think that was a barrier that got in the way of them being able to access that training. So no, there wasn't. Not really

Rachel's account evidenced how organisations which offer peer supporter training may overlook peer supporter needs, indirectly excluding certain populations. Others, including Louise, described their training as accessible, although not focussed on peer supporters' emotional needs:

We did get a little bit [of training] but not a lot really compared to some other organisations. Like, the safeguarding was just an online course that you had to do…It's not necessarily focused around, kind of, looking after yourself and things like that

Louise's statement underlines the lack of training content focussing on peer supporters looking after their own wellbeing. This was echoed by other participants. For example, Richard discussed receiving little to no social support or supervision and the detrimental impact this had on his mental health and emotional wellbeing: ‘The best thing I can think of is […] having someone there with that experience to talk through it and get to the bottom of what went on, what's come up for [peer supporters]’. Richard evidenced the detrimental impact peer support could have on wellbeing and highlighted the importance of discussing stressful events with someone with lived experience to reduce the negative impact on his own mental health and emotional wellbeing.

Jeremy similarly emphasised the emotional impact of peer support work, combined with a lack of social support or supervision: ‘And all that's built up and you take that home and you do get frustrated, but where do you put your frustrations? There's nowhere’. This perceived lack of support left Jeremy feeling isolated and emotionally exhausted. Similarly, Janine felt access to social support was vital in reducing the emotional distress experienced when those she supported relapsed: ‘You see somebody doing well and then they take a step back, and you feel it, don't you? You're gutted for them, and it is, it's definitely having somebody to talk it through’. Janine highlighted the importance of the emotional connection between the supporter and the supported, borne out of lived experience and empathy. She articulated her need to talk to someone about her work supporting others through their difficulties.

Peer supporters who experienced a perceived lack of organisational supervision and social support relied on others for such support. As highlighted by Andrew: *‘*Luckily, I've got a bit of support behind me. I've got a sponsor who I can phone, I've got staff here that I talk to. But some people haven't got that support behind them as volunteers’. On the other hand, Thomas described how his organisation had become less supportive: It's starting to become the sort of thing where they'll say, ‘Oh well, we'll give you a ring in the week’, and ‘then that phone call never comes’. Here, Thomas highlighted how some peer supporters perceived their own support and emotional wellbeing needs to be overlooked by their organisations. This theme illustrated the value of co‐producing training programmes to meet peer supporters' role‐specific and emotional wellbeing needs. Additionally, it highlighted the need to consider peer supporters' lived experiences to ensure they are comfortable accessing training social support and supervision if offered by peer support organisations.

### 3.2 Theme 2: Where does peer support begin and end?

Participants typically described reciprocal, person‐centred support occurring through shared lived experience as the central tenet of peer support provision. However, this theme also evidenced the difficulty of defining peer support and the negative consequences this can have for implementing and upholding boundaries between themselves and those whom they support. Ultimately, this culminated in role ambiguity, with peer supporters having difficulty in establishing and maintaining where their role responsibilities as a peer supporter began and ended. Peer supporter role ambiguity was linked to heightened experiences of stress, abuse and conflict with those they support, often resulting in emotional distress, exhaustion and burnout.

Rachel defined peer support as flexible, person‐centred, and occurring naturally: ‘I think for peer supporters it is what you make it out to be, what is needed at that time for that person’. Rachel describes a person‐centred approach, with peer support being flexible and adaptable to an individual's needs. Such sentiments were also shared by Jason: ‘People get support without knowing it and give support without knowing they are giving support’. Jason also described the benefits of naturally occurring peer support. Extending upon this, Lisa described how formal peer support, akin to professional services, may be intimidating to some:

If it's a professional peer supporter, personally I would want someone to come with a badge. I don't know why. Although you see once you see others with badges, they don't have to read what's on there. You feel like, okay, that boundary is created all of a sudden so you are not at my level

However, ambiguous roles at times led to ill‐defined boundaries, that is, role ambiguity. Jeremy discussed his experiences of role ambiguity and the impact this had on him: ‘Over the last week I've had two phone calls, one on Saturday at around 11:30 to try and help one who was suicidal, and he was threatening to kill himself and then the Tuesday before as well’. Challenges in relation to blurred boundaries between friendship and professional relationships triggered feelings of emotional distress due to a perceived inability to set clear limits within the peer support role. Furthermore, Jeremy described how he had experienced abuse from those he supported: ‘He was fed up and he was leaning on, but when he doesn't get his own way sometimes, when he's drunk, he'll send emails that are quite abusive’. Then, going on to discuss the emotional toll such experiences had on his wellbeing: ‘I do get angry sometimes and you sit at home or in the car and you're like bloody hell, just banging your head against a wall and not getting anywhere and it's just tiring. Yes, it gets quite tiring*’*. Here, Jeremy highlighted the relationship between role ambiguity and emotional exhaustion, due to emotional distress and experiences of abuse and conflict. Such sentiments were also shared by Amanda, who described conflicts occurring in her group's peer support sessions. The individual who had instigated the conflict was asked to leave the group:

We didn't recognise that there were triggers, there were red flags, there were warning flags. […] It was like an abusive relationship; you hope it's going to get better. Somebody says sorry and you think it's going to get better, and it didn't

Such experiences of conflict and abuse heightened peer supporters' emotional distress and exhaustion. As Amanda described, at times, the abuse experienced was akin to an abusive relationship. Amanda further highlighted the negative impact this had on members of the group: ‘That was quite unpredictable, and I think the impact that it had on the group was quite marked in terms of how safe they felt and how secure’. A lack of boundaries facilitated emotionally volatile outbursts which significantly affected peer supporters' own emotional wellbeing. Overall, this theme evidenced how the core values of peer support leave it vulnerable to role ambiguity, making it difficult for peer supporters to implement boundaries. Over time, role ambiguity and lack of boundaries led to peer supporters experiencing emotional distress, and at times abuse and conflict. Experiences of such abuse and conflict led to peers feeling unsafe and emotionally exhausted.

### 3.3 Theme 3: Emotional investment leading to exhaustion

Some peer supporters felt emotionally distressed, exhausted and burnt out due to the emotionally taxing nature of their roles — referred to herein as the emotional labour of providing peer support. This theme also highlights how peer supporters' roles required them to rely upon their own lived experience. However, such experiences often encompassed trauma and emotional distress. This was particularly challenging to peer supporters’ own mental health and emotional wellbeing, especially when exposed to traumatic and emotionally distressing disclosures. Jeremy discussed how peer support could lead to feeling trapped by one's role:

When you are constantly dealing with these kinds of cases, the effect it just, it takes you, it could potentially just take you in a revolving wheel where, you know it feels like there's no way out, but you can't leave your role because you're so important to so many people

Peer supporters discussed becoming emotionally exhausted when the outcomes for those they were supporting remained negative. Paul highlighted the emotional bond between peer supporters and those they supported and described how intensely he felt about deteriorations in others’ circumstances and wellbeing: ‘When you see someone who's totally rock bottom and who's totally given up, and you offer them help and he just doesn't want it anymore. It's quite soul‐destroying if you like’. This degree of emotional investment was linked to Paul's experiences of emotional distress and exhaustion. Thomas told of the emotional impact of hearing distressing disclosures of others' traumatic experiences:

I know guys in there who've lost kids […], like, walked in on their kid hung from the ceiling, and I can't even imagine what that's like for them. So, I think that even though to a degree we have lived an experience, mine does not go that far. And it is hard at those times when you hit that really horrible experience

Peer supporters' ability to cope with these traumatic experiences was sometimes diminished by their own lived experience, not extending to this level of trauma severity. Ultimately, Jeremy felt that having lived experience made peer support provision more difficult:

Having that experience of it, I honestly think that it makes it tougher. Because you put yourself in their situation, you put yourself in their position and that's why you take it home. Now, had you not had those experiences, I think it would be quite easily, easier to just go home, switch off. You don't switch off. I don't switch off ever

Jeremy described how one's ability to genuinely empathise with those they support makes their role more challenging. Consequently, the former is unable to ‘switch off’ from their role, leading to heightened emotional exhaustion and burnout.

Disclosures within peer support could lead to the triggering of memories associated with traumatic events for peer supporters with similar lived experiences, as expressed by Louise:

When they're disclosing stuff, I've found sometimes it can be quite triggering for myself, and then it's meaning that I'm having to take a step back and leave it to other people. Then I feel worse because I'm having to get other people to cover me

Such exposure could lead to deteriorations in peer supporters' mental health. However, Louise went on to describe how peer support also helped maintain her recovery, evidencing the paradoxical nature of peer support: ‘It kind of helps me in my recovery as well, both for my mental health and my addiction, because being able to support others and see them improve kind of encourages me to keep going’. Hence, peer support both aided Louise's recovery and at times hindered it. Accordingly, while shared lived experiences are often integral to fostering positive peer supporter and supportee relationships, depending on the context of the support provided, this can represent significant emotional labour for peer supporters.

## 4 DISCUSSION

This study explored key factors affecting peer supporters' mental health and emotional wellbeing, and opportunities for co‐produced training. Three themes were identified: (1) Insufficient support for peer supporters; (2) Where does peer support being and end? and (3) Emotional investment leading to exhaustion. Collectively, our findings highlight a general lack of peer support‐specific training and the detrimental impacts of this on peer supporters' mental health and emotional wellbeing. Our findings evidence the presence of role ambiguity within peer supporter settings and the negative impact this can have on peer supporters' ability to implement and maintain boundaries, often culminating in experiences of heightened stress and exhaustion. Additionally, our findings highlight the emotional labour of peer support provision and how taxing this can be on peer supporters' own mental health and emotional wellbeing. This study adds to the growing body of literature on peer supporter experiences, highlighting the importance of recognising the mental health and emotional wellbeing challenges that peer supporters face.

Peer supporters discussed the implications of having a lack of role‐specific training and subsequently having to rely on their own lived experience to guide them. Prior research has shown a lack of opportunity for peer supporters to access role‐specific training provided by peer support organisational providers.^13^, ^17^ It has been argued that peer supporter training is paramount to their emotional wellbeing, asserting that training should cover topics such as looking after oneself as a peer supporter due to peer supporters' heightened vulnerabilities in relation to their own mental health.^12^ This was echoed in the current study, with some participants recounting how training did not focus on peer supporters' wellbeing, to their detriment. Our study yielded a novel finding, whereby peer supporters' lived experiences were not taken into consideration. Specifically, female survivors of male‐perpetrated sexual violence were only offered peer supporter training by a male, which proved detrimental to their mental health. As no other training was offered, this subsequently prevented some peer supporters from engaging in training, thus inadvertently excluding a valuable population of the peer supporter community. This novel finding highlights the need to ensure peer supporter training is person‐centred and tailored to their needs, further evidencing the need for co‐produced training programmes in peer support. A lack of support was also identified as a challenge experienced by peer supporters, particularly when combined with the emotional impact, subsequent emotional distress, frustration, and an inability to reduce emotional tension due to lack of support was experienced. It has been found previously that peer supporters often experience inadequate supervision.^6^, ^13^, ^16^ Sufficient supervision was viewed by peer supporters in the present study as integral to maintaining their emotional wellbeing during their roles. This is consistent with recent research highlighting how peer supporter supervision can reduce emotional exhaustion and burnout while concurrently increasing role clarity and job satisfaction.^14^, ^15^

This study also provides further evidence of peer supporter ambiguity regarding the scope and parameters of their roles, while expounding upon the myriad negative implications that role ambiguity can have on peer supporters' mental health and emotional wellbeing. Role ambiguity in peer support is a ubiquitous challenge^10^, ^11^, ^18^ associated with peer supporter dissatisfaction.^15^ In our study, peer supporters defined their role as person‐centred, fluid, reciprocal and naturally occurring through shared lived experience and the desire to help others towards recovery: a description which is broadly consistent with the core principles of peer support.^1^ Here, consistent with previous research,^18^ role ambiguity was associated with a lack of boundaries between peer supporters and those they supported. Peer supporters in this study were sometimes contacted outside of their working hours for various reasons. This included peer supporters being subjected to contact from other peers they were supporting, which they considered to be more akin to friendship, as well as contact where the recipients of peer support disclosed suicidal thoughts and feelings. Ultimately, such instances led to a deterioration in peer supporters' emotional wellbeing, at times presenting a threat to their role maintenance. Elsewhere, role ambiguity has been evidenced as detrimental to peer supporters' self‐care and associated with heightened burnout risk.^12^, ^18^ Our study also identified role ambiguity as contributing towards conflict between peer supporters and those they support, manifesting through abusive language and behaviours. Peer supporters have previously reported a lack of confidence in coping with individuals who present with aggressive and agitated behaviours.^10^ However, to date, there is a lack of research on peer supporters' experiences of conflict and aggressive peers, and the associated impacts on their mental health and emotional wellbeing. Our findings suggest that conflict between peers and their supporters warrants further exploration, to determine its impact on peer supporters' work, and how to best address this issue. Our findings highlight how experiences of conflict and abuse can increase peer supporters' emotional exhaustion, which has been associated with a significant increase in turnover intention.^25^, ^26^

Finally, our study further highlighted the emotional investment leading to exhaustion that peer supporters experience as part of their roles and its detrimental impacts on peer supporters’ mental health and emotional wellbeing. Peer supporters described forming intense emotional bonds forged with those they supported and thus experiencing emotional distress when those they supported encountered negative outcomes such as relapse. Emotional distress and a lack of social support are associated with burnout^14^ and peer supporter relapse.^9^, ^12^ In line with previous literature,^12^, ^13^ peer supporters in our study experienced emotional distress when hearing traumatic accounts. They struggled with role maintenance due to deteriorations in their mental health and emotional wellbeing associated with role stressors and perceived deficits in available support. As such, while shared lived experiences are often considered to form the bedrock of mutually beneficial peer support relationships,^1^ our findings highlight how recurrence of past traumatic experiences triggered vicariously through providing support to others, can present significant challenges to peer supporters' mental health and emotional wellbeing. Additionally, research has evidenced a significant relationship between emotional exhaustion and turnover in peer support, to the same extent as nonconsumer mental health workers.^25^, ^26^ This highlights the need to provide peer supporters with training on emotional wellbeing and access to social support and supervision, to reduce burnout^14^, ^15^ and consequently turnover.

## 5 STRENGTHS AND LIMITATIONS

Peer support research typically focuses on evaluating the effectiveness of peer support interventions for improving recovery outcomes,^3^, ^7^ rather than exploring peer supporters' experiences and challenges and subsequent emotional difficulties which can occur.^24^ Thus, a key strength of our study is that it underscores the importance of understanding peer supporters' training and emotional needs, many of which are not being met. In contrast, a limitation is that we conducted this research within a small, localised area. Evidence suggests peer supporters from smaller localities are at heightened risk of boundary blurring, role ambiguity and burnout,^18^ and therefore, our study findings may not reflect the challenges faced by peer supporters on a national or international level. Thus, our work is exploratory in nature, and further, larger‐scale research is needed, using quantitative or mixed‐method approaches. Notwithstanding, the key themes identified are pertinent to the structures and processes by which peer support is offered. Broadly, while the specific requirements for supporting peer supporters may vary according to specific context‐dependent characteristics, the key themes identified are likely to be applicable.

## 6 CONCLUSION

The study's findings highlight several key challenges in relation to peer supporters' mental health and emotional wellbeing. Moving forward, a key recommendation arising from our findings is for organisations which provide peer support to offer more training opportunities for peer supporters which are relevant to their role, appropriate and accessible. Training opportunities should include guidance and practical strategies for how peer supporters can maintain their own mental health and emotional wellbeing. To ensure such training programmes meet peer supporters' needs, co‐production should be used, which may help reduce the risk of some peer supporters being excluded due to their own lived experience, as found in the present study, and ensure topics such as conflict, abuse and suicidal ideations are addressed, reducing their negative impact on peer supporter wellbeing. Training programmes may benefit from also focussing on defining peer roles clearly and providing suggestions for how to establish and maintain boundaries with those who they will support. Increased access to social and emotional support for peer supporters may reduce feelings of burnout, exhaustion and role captivity. Given the clear benefits of peer support to both the supporter and the supported, it is important that future research consider protective factors to maintain peer supporters' mental health and wellbeing to eliminate or minimise exposure to commonly experienced stressors and the effectiveness of co‐produced training programmes.

## AUTHOR CONTRIBUTIONS

**Laura Kane, Robert M. Portman, Lauren Walker, Emma‐Lily Proctor, Hannah Poulter** and **Catherine O'Neill**: Conceptualisation. **Laura Kane, Lauren Walker, Hannah Poulter** and **Catherine O'Neill**: Data collection. **Laura Kane** and **Lauren Walker**: Data analysis; **Laura Kane, Robert M. Portman** and **Judith Eberhardt**: Writing—original draft preparation. **Laura Kane, Robert M. Portman, Judith Eberhardt** and **Catherine O'Neill**: Writing—review and editing; **Laura Kane, Emma‐Lily Proctor** and **Catherine O'Neill**: Project administration. **Robert M. Portman, Lauren Walker, Hannah Poulter** and **Catherine O'Neill**: Funding acquisition.

## CONFLICT OF INTEREST STATEMENT

The authors declare no conflict of interest.

## Data Availability

The data that support the findings of this study are available from the corresponding author upon reasonable request.
